# Smart Autism Spectrum Disorder Learning System Based on Remote Edge Healthcare Clinics and Internet of Medical Things

**DOI:** 10.3390/s24237488

**Published:** 2024-11-24

**Authors:** Mazin Abed Mohammed, Saleh Alyahya, Abdulrahman Abbas Mukhlif, Karrar Hameed Abdulkareem, Hassen Hamouda, Abdullah Lakhan

**Affiliations:** 1 College of Computer Science and Information Technology, University of Anbar, Anbar 31001, Iraq; abdulrahman@uoanbar.edu.iq; 2 Department of Electrical Engineering, College of Engineering and Information Technology, Onaizah Colleges, Onaizah 2053, Saudi Arabia; saleh.alyahya@oc.edu.sa; 3 College of Agriculture, Al-Muthanna University, Samawah 66001, Iraq; khak9784@mu.edu.iq; 4 Department of Business Administration, College of Business Administration, Majmaah University, Al-Majmaah 11952, Saudi Arabia; 5 Department of Cybersecurity and Computer Science, Dawood University of Engineering and Technology, Karachi City 74800, Pakistan; abdullah.lakhan@duet.edu.pk

**Keywords:** autism disability, autism applications, offloading, scheduling, training, deep convolutional neural network, long short-term memory, smartwatches, mobile and edge nodes

## Abstract

Autism spectrum disorder (ASD) is a brain disorder causing issues among many young children. For children suffering from ASD, their learning ability is typically slower when compared to normal children. Therefore, many technologies aiming to teach ASD children with optimized learning approaches have emerged. With this motivation, this study presents a smart autism spectrum disorder learning system based on remote edge healthcare clinics and the Internet of Medical Things, the objective of which is to offer an online education and healthcare environment for autistic children. Concave and convex optimization constraints, such as accuracy, learning score, total processing time with deadline, and resource failure, are considered in the proposed system, with a focus on different autism education learning applications (e.g., speaking, reading, writing, and listening), while respecting the system’s quality of service (QoS) requirements. All of the autism applications are executed on smartwatches, mobile devices, and edge healthcare nodes during their training and analysis in the system. This study presents the smartwatch autism spectrum data learning scheme (SM-ASDS), which consists of different offloading approaches, training analyses, and schemes. The SM-ASDS algorithm methodology includes partitioning offloading and deep convolutional neural network (DCNN)- and adaptive long short-term memory (ALSTM)-based schemes, which are used to train autism-related data on different nodes. The simulation results show that SM-ASDS improved the learning score by 30%, accuracy by 98%, and minimized the total processing time by 33%, when compared to baseline methods. Overall, this study presents an education learning system based on smartwatches for autistic patients, which facilitates educational training for autistic patients based on the use of artificial intelligence techniques.

## 1. Introduction

According to the World Health Organization [1], the ratio of autism spectrum disorder (ASD) in children has been increasing annually. This disorder leads to a high level of disability in children. Autism is a neuro-brain disease that creates difficulties in children related to learning, reading, writing, communicating, listening, and understanding educational content [2]. Autism is a family-inherited disease that exists among body cells (i.e., dead cells), impacting the child during their childhood. Therefore, significant attention has been paid to autism disease among children, as it is a challenging issue [3].

For further development and progress, many machine learning methods have been introduced to classify autism disease disorder early and predict how to prevent it among children. Machine learning methods have been integrated with autism spectrum disorder (ASD) applications, and may assist children in learning when they suffer from autism. Machine learning approaches can improve ASD systems when they are integrated into educational contexts.

However, all of the solutions mentioned earlier are only based on clinical trials involving MRI, EEG IoT data, and interview session data for ASD detection among children. Therefore, education learning patterns and difficulties among autistic patients have been widely ignored during their treatment at clinical healthcare institutions. Therefore, many research questions related to education and learning for autism-affected children in practice remain yet to be investigated. This study focuses on the following lines of research: (i) The education and learning patterns for autistic patients differ due to their level of disability. Therefore, keeping the balance between trade-off variables (e.g., social disorder, communication skills, learning ability, and understanding with speed) is a challenging task in ASD applications. Therefore, the new system should consider these trade-off variables for educational purposes in autistic patients. (ii) There is no remote autism learning education that collects data in order to better understand the learning patterns among autistic children for the purpose of improving their learning.

This work introduces an IoMT smartwatch-assisted ASD education learning system based on remote edge healthcare clinics for autistic-disabled children. The goal is to introduce a remote learning environment based on smartwatches and edge servers, where all education applications are deployed on the smartwatches. This study makes the following contributions to the considered research problem:

- An IoMT smartwatch-assisted learning system is introduced, in which healthcare clinic stakeholders and educational professionals create the learning environment for autistic disabled children.
- The smartwatch autism spectrum data learning scheme (SM-ASDS) is introduced, which consists of offloading, scheduling, and training schemes for autism applications.
- A combined ASD dataset (e.g., healthcare professional and educationist) is created and trained based on deep CNN (DCNN) and long short-term memory (LSTM) approaches.
- A combinatorial optimizer is introduced to improve the learning efficiency in the context of ASD based on training on the dataset and extracted features on edge servers.

The rest of the paper is formulated in different subsections. Section 2 discusses the related work. Section 3 illustrates the existing ASD systems and their advantages and limitations. Section 4 details the methodology of the proposed system. Section 5 provides an analysis of the experimental results. Section 6 includes the conclusion and future work related to the system.

## 2. Related Work

Autism is a increasingly prevalent disease, and many artificial intelligence tools have been introduced to reduce the level of disability among affected children. Existing autism-related tools, as well as their constraints and methods, are detailed in Table 1. All the studies have integrated IoMT-based applications for autism patients.

The work [2] introduced the emotion-efficient autism system based on IoT deep CNN networks to reduce the disability in communication for autistic children. The DCNN method extracted the features from smartwatch data, monitored their behaviors, and identified their emotions at different clinics. However, this study only focused on the emotional features of autistic patients when communicating with people. A facial expression and recognition-enabled DCNN method was introduced in the work [3], with the objective of extracting autistic features for autistic patients during communication and eye content, in order to improve their level of disability in the environment. However, listening and content understanding issues are critical challenges in the proposed systems. To improve multiple issues associated with autism simultaneously, a federated learning-based DCNN-LSTM method-enabled autism system was introduced in [4]. Many datasets, such as question answers, EEG, MRI, and clinical trials, were used to train the DCNN-LSTM and improve the learning disability of autistic patients in different clinics. The DCNN identified the features of the ASD system, and deep learning methods were introduced to understand the complexities in educational learning among autistic children, allowing for optimal learning and understanding solutions to be generated.

The studies [5,6] introduced IoMT mobile-enabled facial autism systems to detect the disability in children while learning. The training of the autism education learning models—including LSTM and CNN methods—was carried out at remote clinics based on a cloud computing approach. In particular, mobile devices integrate the autism learning interfaces, and their models are trained on edge cloud networks. However, these studies work on only clinical data, and the models cannot be adapted in real-time.

Generative AI, EEG, and IoMT human gesture analysis aware autism systems were introduced in previous studies [7,8,9,10]. The mixture dataset—including brain image, question response, and behavioral data obtained from autistic patients—was analyzed for their learning purposes, and different education tools were introduced. However, the suggested tools suffered from excessive time and resource overheads in mobile and edge cloud nodes during the execution of learning data from autistic patients. Therefore, the overall accuracy needs to be improved with the integration of adaptive learning into these models. The studies [11,12,13,14] suggested that IoMT brain images can benefit autism detection systems with regard to different cases, such as brain disorders, social interactions, and the early detection of language understanding issues in children. Autistic syndrome disorder and other features are considered in these studies based on various datasets, such as the National Database for Autistic Research (NDAR) and the widely exploited Healthcare Research Service (HRS) clinical data. IoT-enabled applications based on local clinical training were introduced in [15,16,17,18,19], with the objective of improving the learning techniques of children with autism disorder. The studies [20,21,22,23,24,25,26,27,28] suggested the use of IoT-assisted autism learning applications based on deep neural networks (DNNs), convolutional neural networks, and transfer learning approaches. The education training model is trained on different nodes based on various features in order to support different autism-related applications, such as gaming, speaking, listening, and so on. However, these techniques have lower accuracy and consume much more time during offloading and scheduling in remote clinics for training after the updating of data.

The studies [29,30,31,32] suggested the use of remote cloud-based autism education therapy and learning skills systems, where the training of workloads is based on the use of a DCNN methodology. Android-based autism applications have been designed, and all training was performed through cloud computing. However, the total time and deadline for these methods are key limitations. The work in [33] suggested surveying autistic patients and discussed the different methods and autism problems in children. However, this discussion was conceptual and theoretical, and more practical testing is required before deployment of the discussed technologies in healthcare contexts. The study in [34] suggested an autism framework for the detection of depression in autistic children during their learning activities from social network data. Different machine learning methods with different parameters were used to train the labeled and unlabeled data for decision making. The study [35] suggested an approach for early autism detection in children based on eye-tracking data. This study exploited a convolutional neural network to extract features from autism data and make a decision based on different features. However, the proposed framework only focused on the communication variables of autistic patients during education learning.

## 3. Proposed Architecture and Methodology

**Figure 1:**
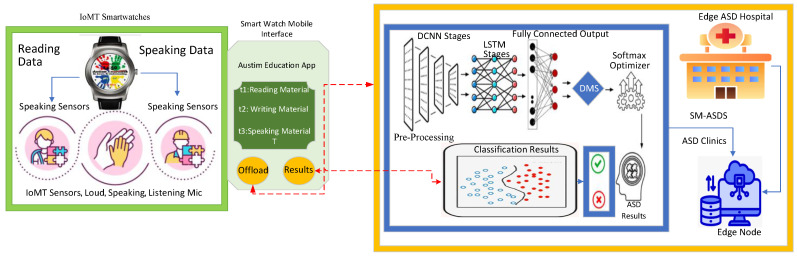
Application and methodology of proposed autism system.

The proposed autism-related system consists of different components, such as applications and methodologies as shown in Figure 1. The proposed system processes the data obtained from autism applications based on the given metrics. The proposed autism system defines all necessary steps of autistic applications connected with non-invasive IoT devices, such as smartwatches, for education and learning purposes. The systematic flows of the proposed system are identified in Figure 1. The proposed system is an adaptive system including many telemedicine applications used at different clinical laboratories as shown in Figure 1. The proposed system performs different operations, such as reading, writing, listening, and speaking for learning goals. This study considers the use of heterogeneous smartwatches, such as wristwatches, mobile phones, and default interfaces. Due to the resource-constrained nature of smartwatches and mobile devices, the system offloads the real-time autism workloads of applications to remote healthcare clinics for processing via communication networks. The IoMT sensors in the smartwatches help to assess different operations such as reading, writing, listening, and speaking.

**Figure 2:**
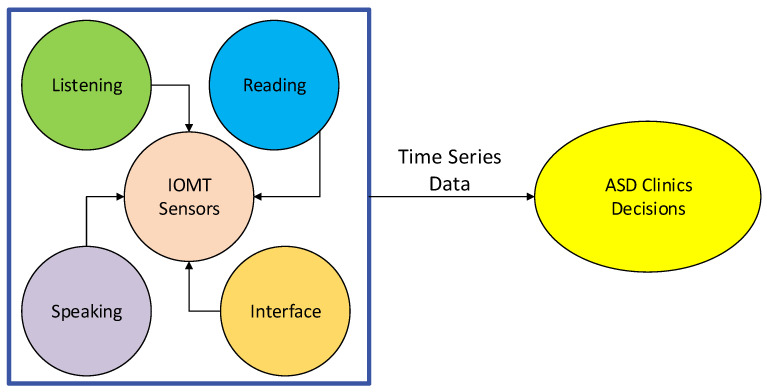
IoMT sensor data offloading and processing.

The system utilizes the IoMT sensors connected with applications for autistic children for learning purposes as shown in Figure 2, where the reading, listening, writing, and speaking sensors are connected with the smartwatches. The ASD applications for reading, writing, speaking, and listening are distributed and connected with remote healthcare clinics, with the goal of monitoring the children with autism and training the learning models on the obtained data. The generated data are quantitatively processed and trained on the clinical servers at a large scale. The smartwatches communicate with the clinical servers based on socket programming for data exchange using the given data standards. Therefore, the smartwatches are only used as an interface, and offload all their data to the clinical servers for training and decision making.

The proposed methodology is shown in the flowchart. For instance, the pre-processing scheme starts processing the offloaded data at edge cloud-enabled clinics. The primary purpose of the pre-processing scheme is to reduce noise and unrelated values from non-linear data before further processing. The data are divided into training, validation, and testing sets in advance, before extracting features from data based on the use of deep convolutional neural networks (DCNNs). The features are extracted based on the pre-processed data in the fully connected layer and hidden dense layer of the DCNN. Our objective is to identify the pattern of educational learning in autism patients. The learning pattern is kept in sequential order, based on the LSTM method, and the optimal learning patterns are obtained to predict future optimized patterns for the autism patients.

A pre-processing technique for analyzing the data based on normalization is suggested. A CNN has different stages, while the ALSTM scheme sequences the data into a particular pattern for usage. The proposed system conducts training on the education data at different clinical nodes using the DCNN and LSTM. In particular, *A* autism applications are considered, where each application *a* includes *T* tasks. In this way, we formulate {a=1,…,A}, with a diversity of polynomial variables and tasks; for instance, {t=1,…,T}. Furthermore, each task has many features (e.g., $t<d_{t},due_{t},s_{t}>$, where $d_{t}$ is the task data, $due_{t}$ is the due time for task *t*, and *s* is the required sensors data for task *t*).

Table 2 provides the notation for all variables and their descriptions. The mathematical model considers *M* mobile devices with distinct resources, such as computing speed $\zeta_{m}$ and storage capability $\epsilon_{m}$. The system considers heterogeneous smartwatch devices *W* with a distinct speed and computing ability (e.g., $\epsilon_{w}$ and $\zeta_{w}$, respectively). The system considers heterogeneous educational healthcare clinical nodes *K* (also with distinct speed and computing ability, denoted as $\epsilon_{k}$ and $\zeta_{k}$, respectively).

The system allows many nodes to execute autism workloads based on their constraints; therefore, the mathematical model facilitates the assignment of different tasks in the following way. Let *x* be the vector variable that indicates the assignment of tasks on different nodes during the processing of autism applications for autistic patients, where x=0 indicates that there is no offloading between smartwatches and mobile devices (i.e., all the tasks are executed on smartwatches); x=1 indicates offloading between smartwatches and mobile devices, where all tasks are offloaded and executed on mobile devices; and x=2 indicates offloading between mobile devices and edge nodes, where all tasks are offloaded and executed on edge nodes, as shown in Equation (1).

Equation (1) describes the assignment of tasks at different nodes when all tasks have different offloading and execution times as shown below:

(1) $$\begin{array}{c} x_{a,t,d,s}=\left\{\begin{array}{cc} x_{a,t,d,s}=0, & \mathrm{Smartwatch}\mathrm{Execution}\\ x_{a,t,d,s}=1, & \mathrm{Offload}\mathrm{to}\mathrm{Mobile}\\ x_{a,t,d,s}=2, & \mathrm{Offload}\mathrm{to}\mathrm{Edge}\mathrm{Node} \end{array}\right. \end{array}$$

(2) $$Watch=\sum_{s=1}^{S}\sum_{a=1}^{A}\sum_{t=1}^{T}\sum_{w=1}^{W}\sum_{d=1}^{D}\frac{a,t,d,s}{\zeta_{w}}.x_{a,t,d,s,w}=0\leq \epsilon_{w}.$$

In particular, Equation (2) determines the time when using only smartwatches during data generation and decision making for autism applications.

The mobile computation time when all smartwatch sensors offload data to mobile devices is determined in the following way:

(3) $$Mobile=\sum_{s=1}^{S}\sum_{a=1}^{A}\sum_{t=1}^{T}\sum_{m=1}^{M}\sum_{d=1}^{D}\frac{a,t,d,s}{\zeta_{m}}.x_{a,t,d,s,m}=1\leq \epsilon_{m}.$$

In particular, Equation (3) determines the mobile computation time during the data generation and decision making for autism applications:

(4) $$Edge=\sum_{s=1}^{S}\sum_{a=1}^{A}\sum_{t=1}^{T}\sum_{k=1}^{K}\sum_{d=1}^{D}\frac{a,t,d,s}{\zeta_{k}}.x_{a,t,d,s,k}=1\leq \epsilon_{k}.$$

Furthermore, Equation (4) determines the edge node time during the data generation and decision making for autism applications.

The following mathematical model determines the data training and validation time:

(5) $$training=\sum_{d=1}^{D}\sum_{s=1}^{S}\frac{Conv[N,d,s].Z[N,d,s]}{\zeta_{k}}\leq \epsilon_{k}.$$

Equation (5) determines the data training time based on DCNN and LSTM on edge nodes, as smartwatches and mobile devices have resource constraint-related issues. Therefore, the system trains the models on the edge node for execution. The training process includes many constraints, such as accuracy, precision, recall, and F1-score. The total processing time of the applications is determined in the following way:

(6) $$Total=\sum_{a=1}^{A}Watch+Mobile+Edge+Training,\;a=1,\ldots ,T.$$

In particular, Equation (6) determines the total time of all applications on all nodes:

(7) Score=Total+Training,a=1,…,T.

Equation (7) determines the autism learning score based on the total processing time and data training for all tasks of different applications. The objective used in this study is determined in the following way:

(8) $$\min Total^{*}\;a=1,\ldots ,T.$$

In particular, Equation (8) determines the optimal total time, leading to higher accuracy of autism learning in all nodes.

## 4. SM-ASDS Algorithm Methodology

**Figure 3:**
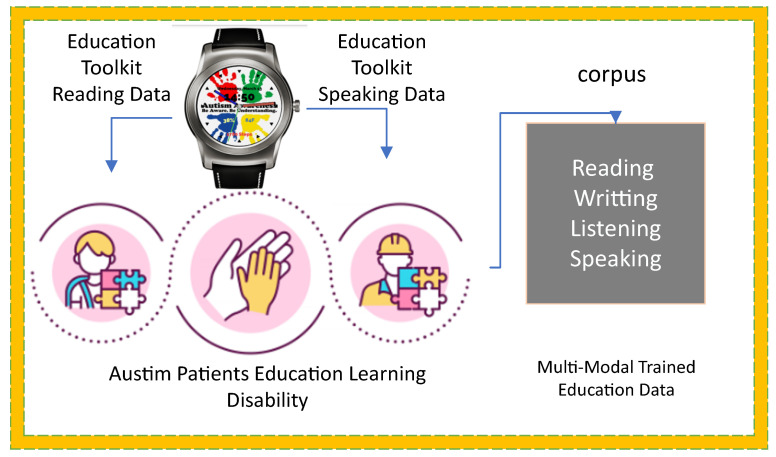
Multi-Modal Data.

The algorithm defines the smartwatch autism spectrum data learning scheme (SM-ASDS) which consists of different methods as shown in Figure 1. The initial trained model of autism education learning uses the limited features integrated with the smartwatches as shown in Figure 3. Each application is integrated as a trained model inside the smartwatches in order to assess speak, write, read, and listen data for educational learning purposes. The natural language processing trained models allow the smartwatch watches to perform various different operations. The proposed SM-ASDS algorithm methodology, including its various sub-methods, is detailed in Algorithm 1; in particular, the sub-methods help to execute the autism patient applications in the system.
**Algorithm 1:** SM-ASDS algorithm.
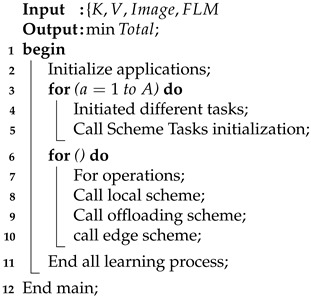


Algorithm 1 shows the SM-ASDS process, which consists of different methods, including local schemes, offloading, and edge cloud-based processing of sensor autism data.

### 4.1. Data Collection from Sensors and Training on Cooperative Devices

**Figure 4:**
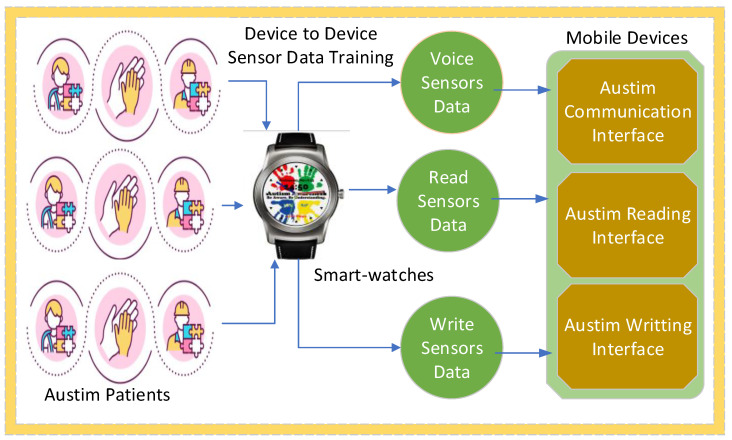
Data processing and training at local smartwatch and mobile nodes.

The algorithm collects data from sensors integrated into smartwatches and mobile devices. Resource constraints are the biggest issue associated with these local nodes. The algorithm introduces pre-compile and processing steps, which are carried out at the local devices to generate and pre-process the sensor data before offloading them to the servers for execution. Furthermore, a novel data training technique that partially trains the data on ad hoc devices to balance resource constraints and computation time is proposed. The algorithm facilitates learning and training scenarios using different devices as shown in Figure 4. The local processing scheme partially trains and processes the sensor data within the ad hoc smartwatches and mobile networks as determined in Algorithm 2. All the steps of the methodology define the steps of Algorithm 2 in the following way:

- The algorithm takes the different parameters (e.g., A,T,S,M,W,D) as inputs.
- All data are collected from different sensors while performing tasks such as speaking, listening, reading, and writing as determined in steps 1–5.
- The data are partially trained on smartwatch nodes and mobile devices, based on the available resources. Therefore, the initial offloading of data is initiated between smartwatches and mobile devices as determined in steps 6–10.
- Local offloading is initiated between local nodes acting as ad hoc networks, where offloading only involves processing the data on local devices, instead of remote offloading to edge nodes.
- All the training actions of autism data sensors are semi-performed on local devices, due to the resource constraints of the devices.
- If the data size imposes high computation and resource constraints, then offloading is performed between local devices and edge nodes for training and execution on data based on their features in networks as determined in steps 10–20.
- All the data models are trained based on combined DCNN and LSTM schemes on different nodes, with respect to the features, time, resources, and deadlines associated with the sensor data.

**Algorithm 2:** Local scheme of data processing and training.
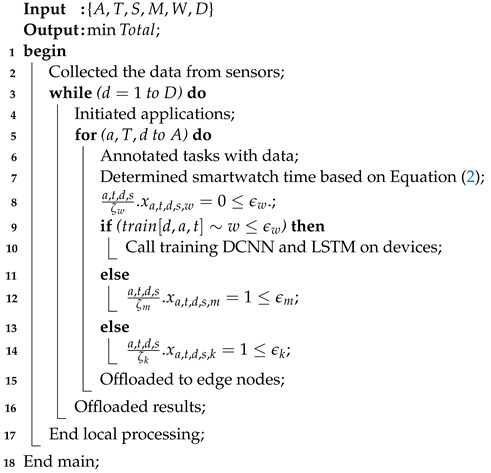


### 4.2. Offloading Scheme

**Figure 5:**
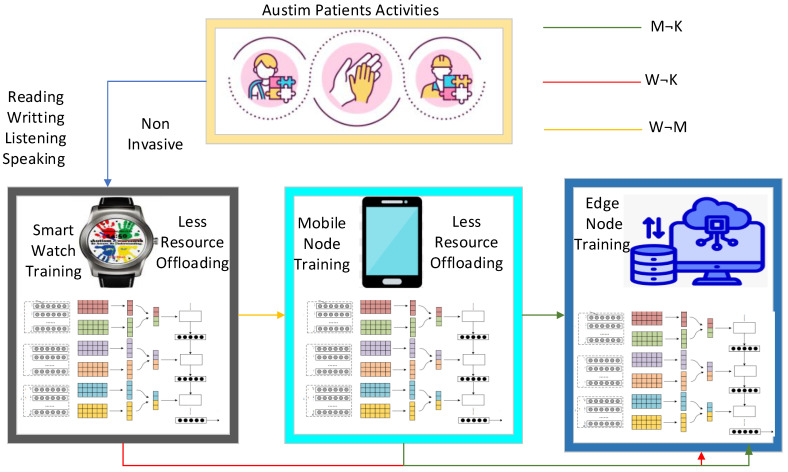
Data training and analysis offloading due to resource constraints.

Offloading is considered the optimal scheme in the considered context, where data are migrated from local devices for training and analysis on different nodes based on the given constraints. The framework includes multiple offloading schemes, involving the migration of data between smartwatches, mobile devices, and edge nodes. Initial offloading occurs between smartwatches and mobile devices to migrate the data and partially train and analyze autism sensor data for education learning as shown in Figure 5. This study considers different applications such as reading, writing, listening, and speaking with different tasks, where the sensors are non-invasive sensors present in smartwatches and mobile applications as shown in Figure 5. All nodes have resource constraint-related issues. Therefore, all sensor data are trained and analyzed on different nodes based on the available resources. As such, an adaptive offloading scheme is utilized, which consists of the different steps detailed in Algorithm 3. Algorithm 3 offloads the generated autism sensor data during learning with respect to different applications, such as reading, writing, listening, and speaking, using different corpuses. In steps 1–12, the algorithm performs the multi-offloading activities based on assignment vectors such as $s,a,t,d,w\leq \epsilon_{w}=0,1,2$. If the vector variable $s,a,t,d,w\leq \epsilon_{w}=0$, the data training and analysis are to be performed on the smartwatch, based on the availability of resources, and similarly for the other conditions. Step 1 shows that the data training and analysis are to be performed on a mobile device, based on the availability of resources, while Step 2 shows that the data training and analysis are to be performed on edge nodes based on the availability of resources. From steps 3 to 12, the offloading and scheduling operations are performed.
**Algorithm 3:** Adaptive offloading scheme.
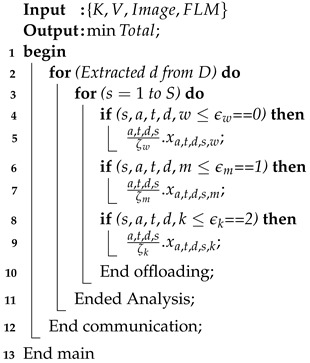


### 4.3. Training DCNN-ALSTM Schemes

**Figure 6:**
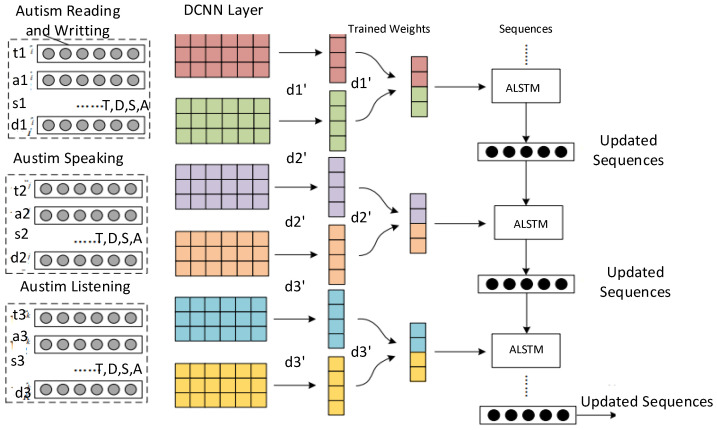
Multi-node training scheme scenario based on DCNN and ALSTM.

Data training and analysis in the heterogeneous autism environment depend upon different hyper-parameters such as the accuracy, learning rate, resource, deadline, and time. The CNN and LSTM methods exploit feed-forward and back-propagation schemes in order to improve the optimization, analysis, and sequencing in the correct order for autism applications. The system includes a multi-node training and analysis scheme based on DCNN and adaptive LSTM (ALSTM) methods. The main idea is that the training of autism patient data on different nodes in partial order minimizes the offloading time, avoids resource failures, and improves the overall accuracy of learning in the network as shown in Figure 6.

Four dataset inputs are implemented in the system for different applications, including reading, writing, listening, and speaking (e.g., t1,a1,s1,d1,w,m,k to T,D,S,A,M,W,K), with different hyper-parameters: deadline, time, accuracy, learning rate, and adaptive sequencing.

### 4.4. Pre-Processing Scheme

The system exploits the linear pre-processing scheme to remove null and useless data values from all the input datasets. The algorithm applies pre-processing before training, and validation on the data is carried out. In the pre-processing stage, normalization is performed, which involves removing the mean zero based on the standard deviation. Similarly, all image sizes in the datasets are made equal. The manual process requires more data modality. Therefore, the algorithm balances the data according to the features of the datasets. Then, a DCNN and ALSTM scheme-enabled scheduling approach is utilized to optimize the different constraints on the autism data during network scheduling as shown in Algorithm 4. Furthermore, Algorithm 4 involves the training and sequencing of the datasets of different sensors and applications in the following way:

- The proposed algorithm is based on DCNN and ALSTM models and includes many stages. For instance, the input layer collects the raw data from different datasets after pre-processing. The convolutional layer is the filter layer, where all features are extracted from different datasets. The algorithm applies the activation layer, where all non-linear data are balanced in particular datasets for the autism classification goal. The fully connected layer connects all channels of the convolutional layer and sends them to the output layer for prediction.
- All datasets related to the sensors based on smartwatches and mobile applications are integrated as inputs from different DCNN channels as shown in Figure 6 and determined in steps 1–3.
- The algorithm uses a CNN to extract features from given datasets in different channels.
- The algorithm exploits a filtering method, which allows it to select relevant correlated features from different datasets.
- For the constraints and dataset feature extraction, the algorithm exploits multi-dimensional word2vec matrices and adjusts their values as defined in steps 3–4.
- The convolutional layer extracts the features, and an update is generated based on the max pooling and fully connected layers as shown in steps 5–10. The constraints of time, resources, data, input gradients, accuracy, and due date are considered hyper-parameters.
- The algorithm splits the trained datasets into different training and testing sets for use on different computing nodes, such as smartwatches, mobile nodes, and edge nodes.
- The sigmoid function evaluates the score of the training model, based on the given inputs and probabilities, and evaluations are made based on the accuracy, time, and due dates of different tasks. These activities are defined in steps 11–12.
- In steps 13–23, the ALSTM adaptive sequence is generated based on the given inputs and processing of the trained datasets. This method is based on the ability of recurrent neural networks to remember the inputs (e.g., of autism data) over time, allowing for the improvement in learning through the sequential orders.
- The proposed method sequences the autism-trained inputs based on a time series, where the learning ratio is improved with different time intervals.
- The input gate takes the trained input weights of autism data, while the forget gate removes those with lower scores in the evaluation by the sigmoid function.
- The output gate is the probability of the sigmoid function, considering whether the obtained score is highest compared to the existing one.

**Algorithm 4:** DCNN-ALSTM multi-node training scheduling scheme.
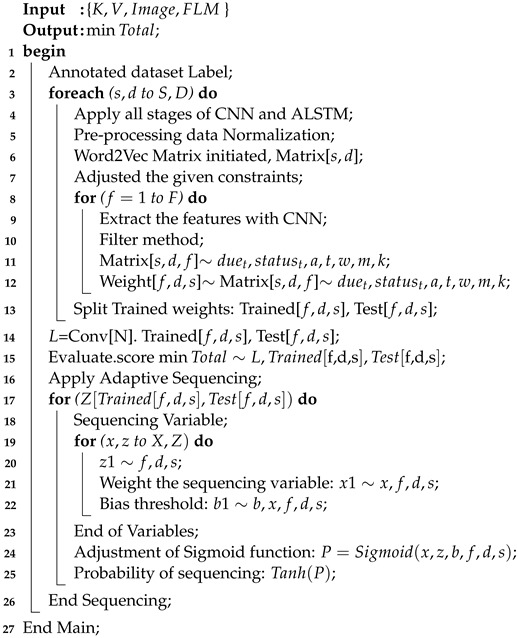


### 4.5. Time Series Computing Scheduler

The time series scheduler is a novel method that schedules the tasks on different computing nodes based on the partially trained models in the networks. The time series describes the sequential offloading and scheduling steps of autism data that are learned and trained on different nodes in the networks within different time intervals.

The timer series scheduler, as shown in Algorithm 5, partially starts the execution process to improve the overall learning rate of autism data in different time series. Partial training denotes the process of training, testing, and validating the autism datasets on other nodes with differences in divergence and convergence. Each node (i.e., smartwatches, mobile devices, and edge nodes) can train and analyze the autism data based on the available resources and given task deadlines in the networks.

The system defines the time series scheduler in Algorithm 5, including the different steps:

- The time series method has different stages, such as input, forget, and output gates, which are connected with a convolutional neural network. The convolutional neural network inherits the input layer, comprising the words in the sequences from different time services. Meanwhile, the forget state learns from the last, and the output layer outputs the sequence data in the system.
- In steps 1–8, the scheduler allocates all tasks to the smartwatches, which partially train the autism data based on DCNN and ALSTM models within their resource capabilities and given the deadlines of tasks for different applications.
- In steps 9–13, the scheduler allocates all tasks to the smart mobile devices, which partially train the autism data based on DCNN and ALSTM models within their resource capabilities and given the deadlines of tasks for different applications.
- In steps 10–20, the scheduler allocates all tasks to the smart edge nodes, which partially train the autism data based on DCNN and ALSTM models within their resource capabilities and given the deadlines of tasks for different applications.

**Algorithm 5:** Time series computing scheduler.
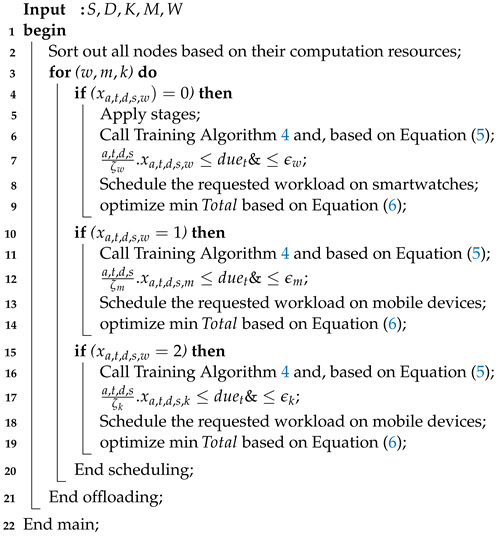


The scheduler in Algorithm 5 allocates all tasks of different applications to the available resources until all the results are generated positively and with minimum total and higher accuracy in the network.

## 5. Performance Evaluation

In this part, the results of experiments on different scenarios with diverse autism datasets and applications are detailed. The evaluation includes the simulation configuration, implementation, dataset description, result analysis, and findings and limitations.

### 5.1. Autism Simulation Configuration

The simulation environment consisted of various parameters from development to implementation, as shown in Table 3. In particular, the simulation environment for the autism data was the same as that deployed in [36]. The algorithm exploited languages such as JAVA for application programming interfaces, Python for data training and validation, and Kotlin for application development in the Visual Studio environment, which supports cross-platform technologies for autism applications. The proposed platform was deployed in a high-performance computing machine for the simulation experiments.

The devices, such as smartwatches, mobile devices, and edge node configurations, are also provided in the simulation file as shown in Table 3.

### 5.2. Autism Disability Simulator Implementation with Dataset

**Figure 7:**
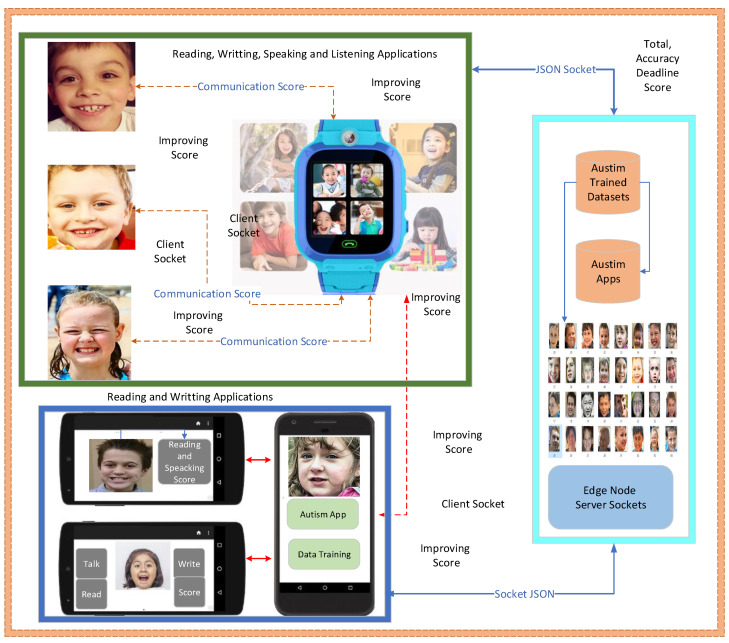
Autism disability simulator implementation.

In the simulation, the experimental environment was designed based on clinical ASD applications which interface with mobile and smartwatches and are connected to edge hospitals as shown in Figure 7. The system integrated the balanced datasets including sensors data as shown in Table 4 with the applications as detailed in Table 5.

The implementation part consists of many applications, including those related to reading, writing, speaking, and listening, with a diversity of tasks. The tasks include registration, login, identification number generation, portal data, learning schedule, course content, video streaming, file uploading and downloading, and many more. The system develops this implementation of autism based on socket programming, where different local devices and edge nodes can use the cross-platform X86 environment for communication and data sharing, thus enabling training and learning in the networks. The system allows for the connection of IoMT sensors related to digital healthcare and extended versions of IoT sensors. Generally, IoT sensors are more generic and used in a wide range of applications. However, the proposed system only focuses on IoMT sensors related to the medical domain, particularly used for autistic patients during learning. The score is determined by offloading and scheduling communication, reading, writing, and listening content from applications based on the behaviors and activities of autism-disabled children. The autistic children can exploit their IoMT devices as sensors (e.g., voice sensors), alerts, notifications, alarms, and speaking sensors directly attached to the devices, which are treated as the data for processing and training in our simulation as shown in Figure 7. Therefore, the learning environment is adaptive, allowing different schools or academic institutions to train their learning models on clinical edge servers in order to optimize the learning patterns of disabled children, as shown in Figure 7. All the devices, IoT smartwatches, and IoT mobile devices of autistic patients are connected to remote healthcare clinics to learn, monitor their learning progress, and evaluate their obtained scores in the environment for improvement. Each computing node can train and validate the dataset; however, training and analysis in parallel computing are only performed on edge nodes due to their resource capability and storage constraints.

The framework was implemented with a variety of different sensors in the simulator. In particular, smartwatches are virtual simulated nodes with mobile edge clouds for education applications for autistic children as detailed in Table 4. In a virtual smartwatch designed for children with autism spectrum disorder (ASD), multi-modal data collection technological solutions should ideally incorporate a variety of sensor types for multi-modal reading, writing, speaking, and listening analyses. The framework employs microphones with speaking and listening, which record audio data to evaluate the child’s verbal response and listening skills as part of a holistic design targeting speaking, listening, reading, and writing abilities. Accelerometers and gyroscopes are used to monitor the direction of writing and reading behaviors, which are frequently linked to ASD. Furthermore, proximity sensors can show how closely a child interacts with printed or screen-based content, while eye-tracking eye movements during reading, writing, speaking, and listening can measure focus and attention span to identify reading engagement. Smartwatches also include gyroscope sensors which can be used for frequency and amplitude analysis, including speaking-, writing-, and reading-related motion signals. These sensors are integrated into a virtual smartwatch that generates data for ASD interfaces; the system needs to include thorough descriptions of all sensors, signal characteristics, and processing algorithms intended for real-time multi-modal monitoring. As the multi-modal data collecting component is essential to develop a responsive and encouraging wearable device for ASD therapies, it might be challenging to verify the smartwatch’s efficacy in children with ASD without these components.

### 5.3. Dataset Description with Applications

The system collected a dataset based on IoMT sensor activities and exploited an existing trained model for autism patient detection and education learning based on machine learning [4,37]. In the simulated implementation, the system focused on the learning ratio of autistic patients, rather than detecting the autistic patients. The system collected the different activities during the educational learning of autistic patients as detailed in Table 5.

The score is a variable that determines the highest accuracy of data learning when ASD applications perform multi-modal data operations on smartwatches and edge cloud nodes, as determined in (7). The score is higher when all tasks (e.g., speaking, reading, listening, and writing) are performed accurately and executed on the nodes (e.g., smartwatches and edge cloud) at the given time. Therefore, the score variable is defined as the optimal constraint of the ASD framework, which improves the learning capability of ASD applications and frameworks while performing multi-modal operations for ASD children. For the score calculation, the system trains the model in advance with respect to the considered operations (e.g., reading, listening, writing, and speaking). The ASD application offloads the data from smartwatches at runtime, and the score is calculated at runtime during learning data processing in the framework.

The dataset consisted of hyper-parameters such as application, parameter, feature, language, and length (MB). The parameters were integrated into the data collected from different sensors. The dataset used for the system is shared in a public repository for use in further research. The system also used an existing dataset with different parameters as detailed in Table 6.

The algorithm proposed in our previous work [4] obtained the optimal results to detect autistic patients with learning disabilities; meanwhile, the algorithm proposed in this study allows their learning abilities to be improved.

The considered datasets consist of various modalities and were tested separately. In particular, separate testing was performed to justify the superiority of the proposed method as detailed in Table 7.

### 5.4. Discussion

Different baseline approaches were utilized in the simulation, and the results were compared with those of existing methods to better assess the results. The experiment integrated different methods, including SM-ASDS, ASD-SM, ASD-IoT, and ASD-Edge [11,15,17,20,21,24,25,26,27,28], in order to evaluate the performance of IoMT applications for education learning with different constraints. These baseline methods are integrated into the discussion for comparison of the results. Based on the obtained results, the performance of the proposed method is discussed in comparison with the baseline approaches.

The system implemented different statistical methods, such as ANOVA, considering many constraints such as time, learning rate, accuracy, score, time, deadline, and resource failure in the simulation results. Statistical analysis and interpretation of the results were exploited in order to discuss how they were derived and their implications for autism education and IoMT using the different methods. Therefore, all the results were compared based on the statistical methods and constraints mentioned earlier.

Table 8 provides the performance metrics for the SM-ASDS, ASD-SM, ASD-IoT, and ASD-Edge methods, with the metrics being based on statistics defined in the paper [38]. The simulation allowed for a comparison of the performance of all applications based on different methods for concave optimization, as shown in Table 8. The accuracy, recall, precision, and F1-score metrics were considered for the application methods in the evaluation. The performance indicators in Table 8 demonstrate that SM-ASDS obtained the optimal results in terms of accuracy, recall, precision, and F1-score, when compared to the existing methods.

**Figure 8:**
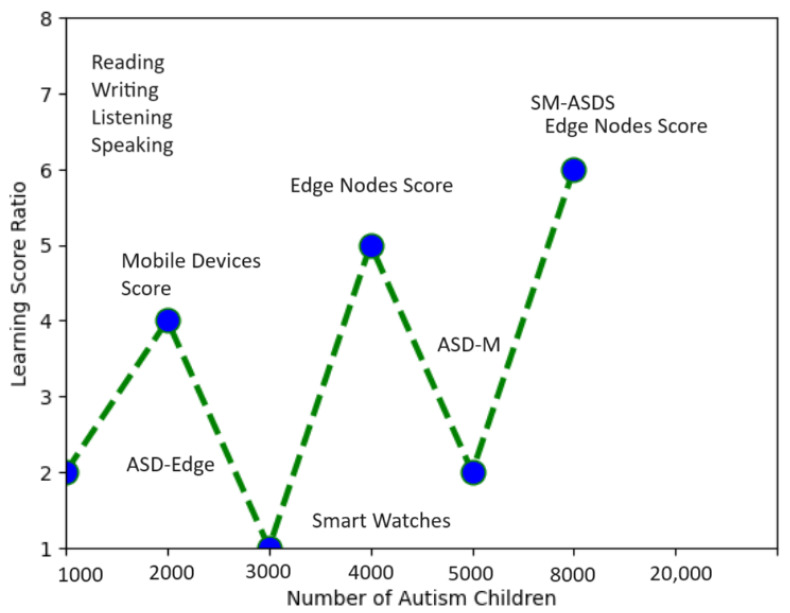
Learning score from analysis.

**Figure 9:**
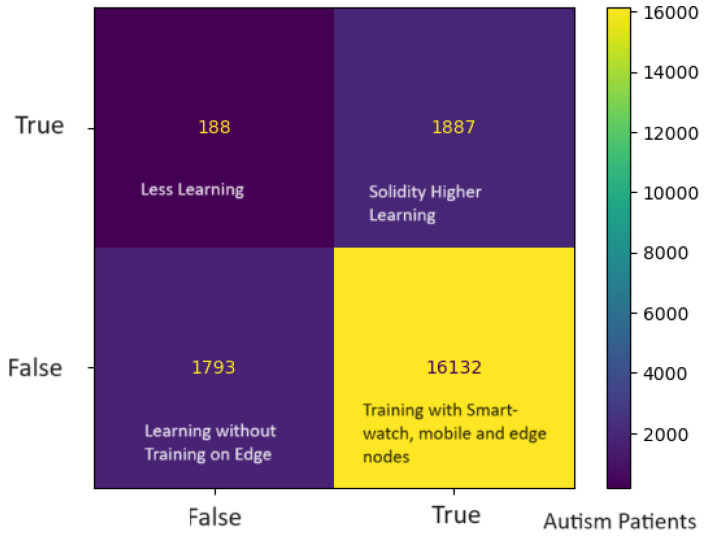
Confusion matrix for learning on different nodes.

**Figure 10:**
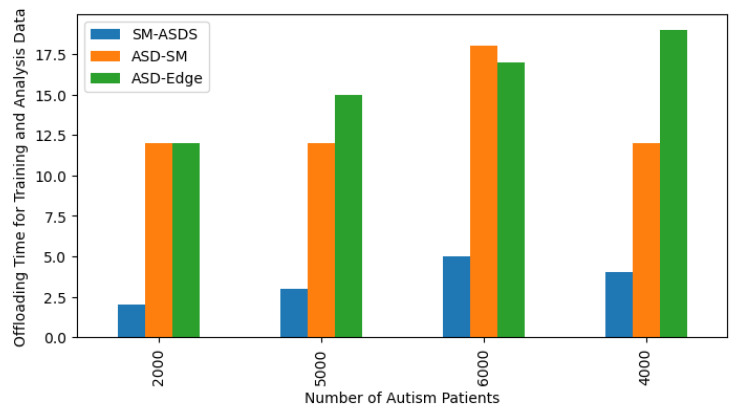
Offloading time during training and analysis.

Figure 8 shows the higher learning score ratio on the trained data when different nodes were used for analysis using the proposed scheme. The highest score is considered a statistical constraint for comparing IoMT education learning methods. In the scenarios of reading, writing, speaking, and listening applications, the learning score results indicate that autistic patients were learning more or still learning, but to a lesser extent, under the adapted simulator patterns. A total of 20,000 autistic children were involved in the learning phase, who presented different learning skills with different nodes, such as smartwatches, mobile, and edge nodes. Due to the resource-constrained nature of smartwatches, the learning and training mechanism had a lower score when compared to mobile and edge nodes, as shown in Figure 8. The proposed SM-ASDS method outperformed all other methods, including ASD-M, which is implemented in smartwatches and mobile devices, and ASD-edge, which implements edge nodes for autism data processing. The proposed system exploits DCNN and LSTM models for pattern detection and sequencing of the words in the complex autism data used in the simulation. The system detects different patterns, such as learning with more repetition practices, saying words repeatedly, and speaking with errors and error-free speaking as part of the training approach. The confusion matrix is shown in Figure 9, where a total of 20,000 autistic children who learn with different patterns were analyzed to evaluate their learning scores as statistical constraints, in terms of false or true terms. The simulation results, as shown in Figure 10, indicate the offload time-related performance when the patient uses an application. As defined in Figure 10, the SM-ASDS presents the optimal offloading times when compared to ASD-SM and ASD-Edge, suggesting a more effective processing system in the SM-ASDS framework. However, a more thorough understanding of the system’s consistency might be obtained by looking at the standard deviation of offloading times between patients. A lower standard variation in offloading times across patients would indicate a more stable and predictable system, indicating that SM-ASDS maintains consistent performance independent of patient-specific factors. On the other hand, a higher standard deviation could suggest that the offloading time variability of the system is influenced by patient-specific characteristics such as mobility patterns, data intensity, or network circumstances. Therefore, the standard deviation indicates the system’s efficiency when processing patient data, with respect to the diversity of the system. Due to the resource-constrained nature of smartwatches and mobile devices, it is suggested that the offloading method is used to improve the training and validation of the model of autism data on different edge nodes. The system integrates three methods—SM-ASDS, ASD-SM, and ASD-Edge—which are compared in terms of offloading of autism data to various nodes. The simulation results compare the offloading time, which directly impacts the learning performance of autism scores through model training and analysis on other nodes. Figure 10 shows that SM-ASDS has a lower offloading time than ASD-SM and ASD-Edge. The main reason for this is that SM-ASDS is partially trained on different nodes, which minimizes the overall offloading time and processing time associated with statistical constraints, such as those of ASD-SM and ASD-edge, which incur higher offloading time during autism data learning in the networks.

**Figure 11:**
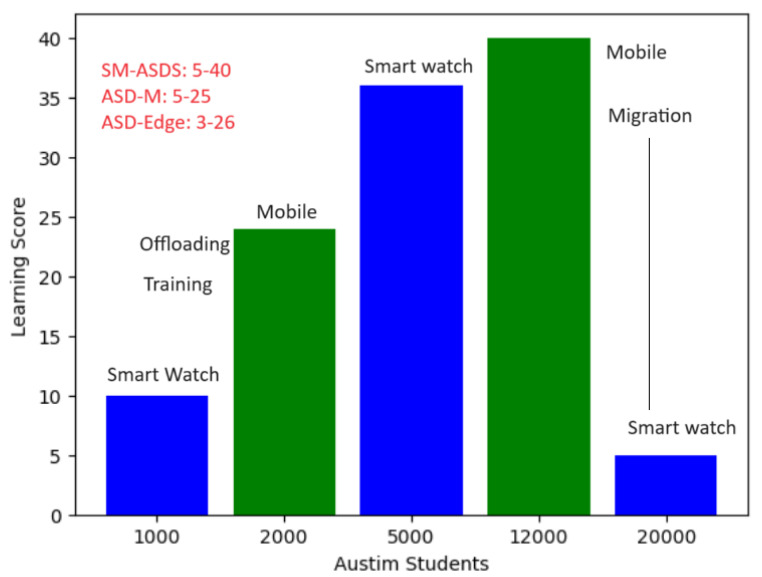
Multi-offloading partial learning score from training and analysis.

**Figure 12:**
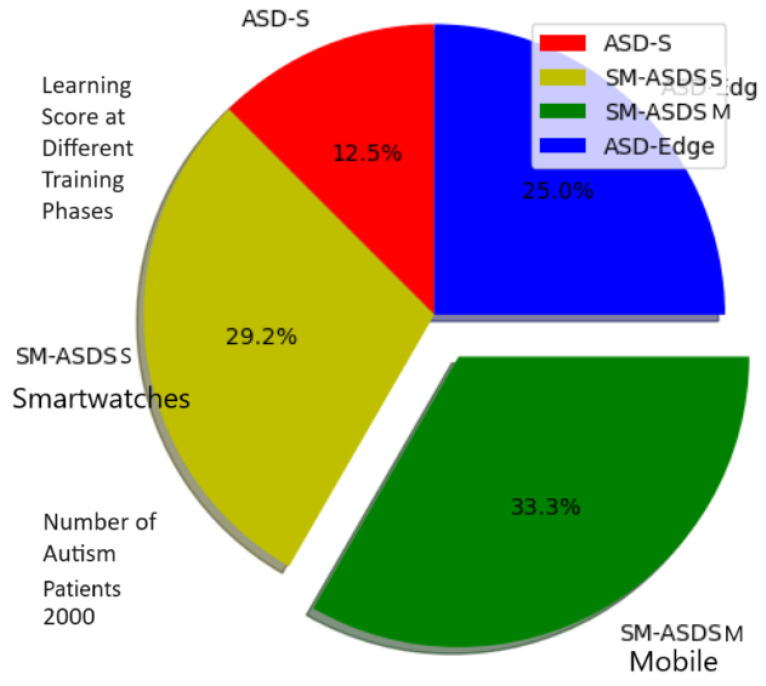
Cooperative scenario of score learning on different cooperative nodes.

**Figure 13:**
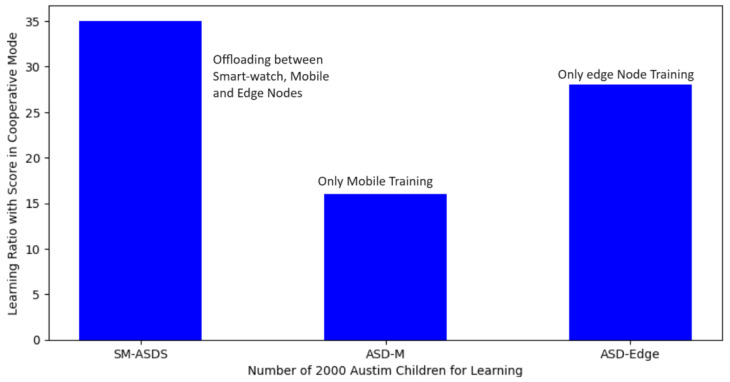
Without Cooperative Score Learning on Different Nodes.

**Figure 14:**
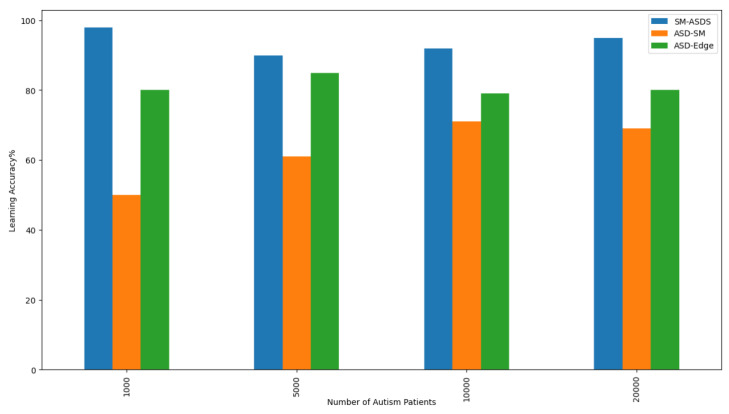
Accuracy of learning and data processing under different numbers of autism patients.

In Figure 11, the green color shows the learning score, including processing time, data generation, and learning on the smartwatch, mobile, and edge cloud nodes. The learning score performance results are compared during execution and offloading between smartwatches and other nodes as shown in Figure 11. The blue color node identifies the performances of smartwatches in terms of the score (as shown in the y-axis in Figure 11). Meanwhile, the green graph bar shows the learning scores of other nodes during the execution of multi-modal operations after offloading. Figure 11 shows that partial learning among different smartwatches, mobile devices, and edge nodes leads to higher learning scores in terms of the statistical constraints with the SM-ASDS, when compared to ASD-SM and ASD-Edge, during the execution of autism applications. The main reason is that partial learning allows different tasks to be performed with less analysis time and processing time through offloading to different local nodes, such as smartwatches and mobile nodes. Figure 11 shows that SM-ASDS has a higher ratio of learning and improvement for all applications with different tasks, when compared to the existing methods.The learning score in cooperative environments, such as that using smartwatch, mobile device, and edge cloud nodes, could be improved. The results for SM-ASDSS indicate that the learning score during training, execution, and offloading could be improved through cooperation with other nodes, such as SM-ASDSM, as shown in Figure 12. Therefore, the different scenarios reveal that, in a cooperative environment, the workloads of smartwatches for multi-modal operations could be improved on different nodes with the SM-ASDS algorithm utilized in the framework. Figure 12 shows that the cooperative learning pattern on single nodes and the offloading of coarse-grained workloads to the edge nodes led to less-effective results for all applications; meanwhile SM-ASDS-trained autism task workloads on different cooperative nodes made decisions based on optimal offloading and total time, and maximized increased scores, when compared to the existing methods.Figure 13 shows that, without cooperative learning patterns among different nodes, executing the entire workload of applications on single nodes led to less effective results for all applications. Figure 13 also shows that, by training autism workloads on different cooperative nodes, SM-ASDS made decisions based on optimal offloading and total time, thus maximizing the obtained scores, when compared to those without cooperative strategies in the simulation. In Figure 14, it is shown that SM-ASDS effectively used the standard deviation in time scheduling across various scenarios, demonstrating its resilience and consistency. The patient’s standard deviation relates to learning, speaking, listening, and writing activities in autism applications. Therefore, using the standard deviation single ANOVA method, SM-ASDS outperformed the compared techniques in terms of learning scores and achieving task deadlines. The low variation validates the efficacy of SM-ASDS in dynamic scheduling situations, consistently satisfying the time and accuracy goals for patients with different features. Through examining the consistency and mean performance of SM-ASDS, this investigation strengthens the argument for its superiority. If the standard deviations are available or can be calculated, their addition would serve to strengthen similar methods.

The results indicate the learning accuracy of autistic students based on the learning scores and minimum total processing time. In this context, accuracy is the trade-off between the learning score and the minimum total time during talking, speaking, listening, writing, and reading applications of autistic patients in the system. Figure 14 shows that SM-ASDS had higher accuracy, in terms of the learning score, and scheduled all tasks with the minimum total time and within their deadlines, compared to the other approaches. SM-ASDS obtained 98% accuracy, higher than that of the existing methods. The main reason for this is that SM-ASDS offloads and schedules all tasks using the adaptive patterns, while learning how to promote increased scores in the simulation.

**Figure 15:**
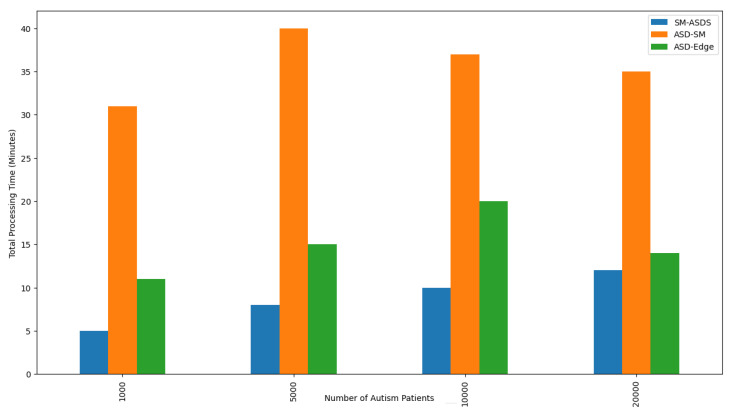
Total processing times on smartwatch, mobile, and edge nodes.

The algorithm partitioned the processes of different applications into different nodes, minimizing the overall total time of data training and analysis. Figure 15 shows the total processing time of all autism applications during their training, offloading, and scheduling on different nodes during execution in the networks. Figure 15 shows that SM-ASDS had 33% less processing time, when compared to the existing baseline approaches, due to partitioning, offloading, and scheduling on different nodes. In particular, the process consisted of offloading, training, testing, and scheduling on different nodes based on a given deadline, resource, and time constraints during execution.

**Figure 16:**
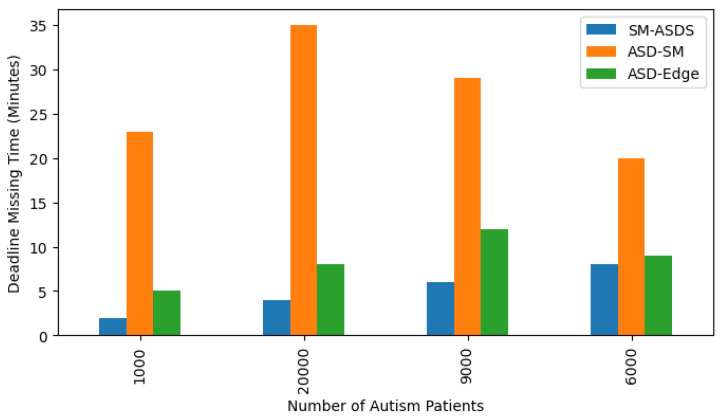
Deadline failure processing times on smartwatch, mobile, and edge nodes.

Figure 16 shows that SM-ASDS had a lower deadline failure ratio, when compared to existing methods, due to the partitioning of applications for training and analysis in the heterogeneous smartwatch, mobile, and edge nodes. Each application is characterized by different tasks (e.g., accessing data, speaking speed, reading online, writing online with score feedback), with each task having a deadline. Figure 16 shows that SM-ASDS outperformed and had fewer deadline failures than existing methods. The algorithm in the proposed system considers both concave and convex optimization, where the deadline of tasks is critical during offloading and scheduling on different nodes in the system.

**Figure 17:**
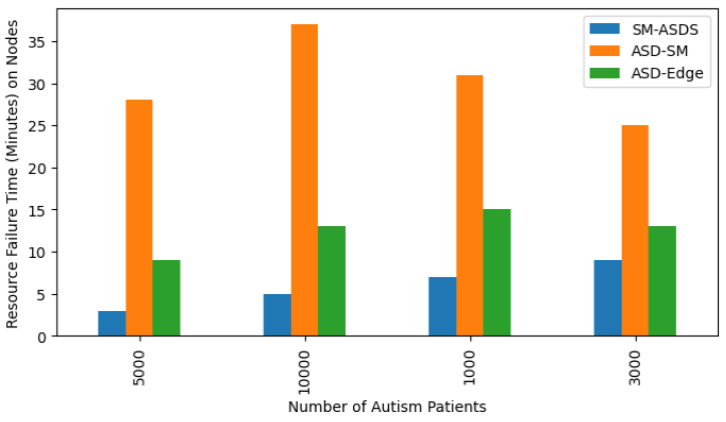
Resource failure processing times on smartwatch, mobile, and edge nodes.

Autistic children can use applications with limited services, such as smartwatches, allowing only a small amount of training based on their available resources. Therefore, offloading is required to extend the services on the different nodes for training with diversity, such as mobile computing, serving as cooperative nodes in the system. However, smartwatches and mobile nodes are resource constrained, and more resources are needed for data training and offloading the workloads of autism applications to edge nodes through various communication networks. This can lead to different resource failures and missing task deadlines on different nodes. SM-ASDS minimized the deadline failure of all tasks as shown in Figure 16. Figure 17 shows that SM-ASDS had a lower resource failure ratio, when compared to existing methods, due to the partitioning of applications for training and analysis in the heterogeneous smartwatch, mobile, and edge nodes. Each application requires a certain amount of resources, and the proposed system partitions the applications according to their available resources. Figure 17 shows that SM-ASDS outperformed the existing methods, having fewer resource failures overall.

### 5.5. Findings and Limitations of Proposed Method

The main finding of this study is that the proposed learning system for autistic children, which is based on the use of smartwatches in a distributed environment, presents evidence of significant improvement in learning when autistic children use this education IoMT application, thus improving their capabilities. During testing, the proposed system was characterized as having the highest accuracy, learning score, and minimum processing time, and fulfilled the deadlines for IoMT applications. However, there are still many limitations to the proposed system; for example, the proposed system is distributed in nature and, so, there must be a security mechanism to protect the healthcare data in different clinics and networks. Therefore, security components must be added to the system to ensure the safety of data and network validation.

## 6. Conclusions and Future Work

In this study, an educational system for Autism spectrum disorder (ASD)-affected children was presented. In particular, a Smartwatch-Assisted ASD Learning System based on Remote Edge Healthcare Clinics for Children with Autism Spectrum Disorder was introduced. The study defined the aim of the system and presented the educational learning system for autistic children. The system involves smartwatch-level autism education applications, which are then executed on mobile devices and edge healthcare nodes in the training and analysis phases. The proposed system—as shown in Figure 1, which shows the entire application process—offers different services to autistic patients during learning and shares the obtained data with associated hospital nodes. This study presented the smartwatch autism spectrum data learning scheme (SM-ASDS), which comprises various offloading approaches, training analyses, and schemes. The SM-ASDS algorithm methodology incorporates partitioning offloading, deep convolutional neural network (DCNN), and adaptive long short-term memory (ALSTM) models to facilitate training on autism data across different node types. The simulation outcomes demonstrate that SM-ASDS obtained enhanced learning scores (by 30%), accuracy (by 98%), and diminished total processing time (by 33%), when compared to baseline methods.

However, when considering the remote healthcare learning process, cost and resource constraints are the main issues that existing systems face. In future work, the autism system will be optimized through the inclusion of cost constraints for many more applications.

## Figures and Tables

**Table 1:** Autism detection and learning tools.

Paper	Application	Method	Objective	Node
[2]	IoT Emotion	DCNN	Prediction	Cloud
[3]	IoT Facial	DCNN	Prediction	HPC
[4]	IoT Hybrid	DCNN-LSTM	Time, Accuracy	Edge-Cloud
[5,6]	IoT Facial	Mobile CNN	Accuracy	Cloud
[7,8,9,10]	IoT Human Gesture	CNN-LSTM	Accuracy	Cloud
[11,12,13,14]	IoT Brain	CNN	Accuracy	Edge
Proposed	IoMT Learning	SDCNN-LSTM	Hybrid Constraints	Edge

**Table 2:** Experiment configuration.

Symbol	Description
*A*	Number of applications
*a*	Particular application
*T*	Number of tasks of application *a*
*t*	Particular task
$due_{t}$	Due date of task
$status_{t}$	Status of task
*S*	Number of consumer sensors
*s*	Particular sensor
*D*	Multi-modal data of sensors and system
*d*	Distinct data of sensors or system
*W*	Number of smartwatches
*w*	Particular smartwatch
$\zeta_{w}$	Speed of smartwatch
$\epsilon_{w}$	Resource of smartwatch
*M*	Number of mobile devices
*w*	Particular mobile device
$\zeta_{w}$	Speed of mobile device
$\epsilon_{w}$	Resource of mobile device
*K*	Number of Clinical nodes
*w*	Particular clinical node
$\zeta_{w}$	Speed of clinical node
$\epsilon_{w}$	Resource of clinical node
Conv[N]	Convolutional layers
Z[N]	Longer memory learning rate

**Table 3:** Parameters of the simulation experiment.

Values	Description
Languages	Socket JAVA
Training	Python
Application	KOTLIN
Environment	Android X86
Angel-Sense smartwatch	1 GB ROM, 1 GB RAM, 2000
Samsung Android	64 GB ROM, 1 GB RAM, 2000
Edge Nodes	1 TB∼5 TB ROM, 128∼256, 4
Convolutional Layer	16×16
LSTM Gates	3
Hidden Layer	32
Analysis method	ANOVA Multi-variance
Experimental Repetition	198

**Table 4:** Details of multi-modal sensors used in smartwatches designed for children with ASD.

Sensor Type	Functionality	Multi-Modal Device
Microphones	Speaking and listening	Smartwatch
Accelerometers	Monitor direction and movement	Smartwatch
Gyroscopes	Reading and Talking	Smartwatch
Proximity Sensors	Writing and Reading screen content	Smartwatch
Eye-Tracking Sensors	Speaking and Responding	Smartwatch
Infrared Sensors	Reading and Write	Smartwatch

**Table 5:** Autism learning dataset.

Application	Parameter	Feature	Language	Length (MB)
a1:Reading	Total, Score	Characters	English	100 MP4
a2:Write	Total, Score	Characters	English	500 MP4
a3:Communication	Total, Score	Voices	English	1000 MP3
a4:Listening	Total, Score	Voice, Video	English	500 MP4

**Table 6:** Results of training the method proposed in [4] on autism application dataset.

Dataset	Method	Accuracy	Recall	Precision	Application
ASQ	ASD-CNN	92	0.94	0.96	ASD
CSBS	ASD-CNN	95	0.93	0.92	ASD
PEDS	ASD-CNN	96	0.92	0.91	ASD
EEG	ASD-CNN	93	0.95	0.93	ASD
Gaming	ASD-CNN	92	0.94	0.96	ASD

**Table 7:** Dataset training, validation, and testing.

Dataset	Application	Parameter	Feature	Size (MB)	Node
**Training Dataset**					
a1:Reading	Total, Score	Characters	English	100 MP4	Edge
a2:Write	Total, Score	Characters	English	500 MP4	Edge
**Validation Dataset**					
a3:Communication	Total, Score	Voices	English	1000 MP3	Edge
**Testing Dataset**					
a4:Listening	Total, Score	Voice, Video	English	500 MP4	Edge

**Table 8:** Performance evaluation metrics.

Dataset	Method	Accuracy	Recall	Precision	F1-Score	Application
ASQ, CSBS, PEDS, Gaming	SM-ASDS	98	0.98	0.98	0.97	ASD-Education
ASQ, CSBS, PEDS, Gaming	ASD-CNN	95	0.93	0.92	0.93	ASD-Education
ASQ, CSBS, PEDS, Gaming	ASD-SM	96	0.92	0.91	0.92	ASD-Education
ASQ, CSBS, PEDS, Gaming	ASD-Edge	93	0.95	0.93	0.92	ASD-Education
ASQ, CSBS, PEDS, Gaming	ASD-IoT	92	0.94	0.96	0.91	ASD-Education

## Data Availability

The study utilized existing datasets published in our previous work [4], with the following details. The implemented datasets included those such as the Ages and Stages Questionnaires (ASQ), Facial Communication and Symbolic Behavior Scales (CSBS), Parents Evaluate Developmental Status (PEDS), modifying Checklist for Autism in Toddlers (M-CHAT), and Screening Tool for Autism in Toddlers and Children (STAT) datasets, which were processed in different computing laboratories and are publicly available from the following link: https://github.com/Abdullah-Lakhan/ASD-Code-and-Datasets/tree/main (accessed on: 11 December 2024). The code for the project is also available at the same link, which has already been used in publications regarding the same project (i.e., autism applications).
